# ADDRESSING OLDER ADULT SOCIAL ISOLATION AND ABUSE THROUGH RURAL OUTREACH AND INTER-ORGANIZATIONAL COLLABORATION

**DOI:** 10.1093/geroni/igz038.229

**Published:** 2019-11-08

**Authors:** Naomi R Meinertz, Megan Gilligan, Jeongeun Lee, Louise Peitz

**Affiliations:** Iowa State University, Ames, Iowa, United States

## Abstract

Elder abuse is commonly linked with social isolation and in a rural state, such as Iowa, older adults may be at increased risk of social isolation and elder abuse. A community-based needs assessment aimed to record the first-hand perspectives of service providers regarding the needs of older adults in rural areas across the state of Iowa, covering 54 of the 99 counties. Through a survey (N=202) and focus groups (N=24), service providers, including direct care, Area Agencies on Aging, law enforcement, and attorneys, offered ways in which to address the gaps in service provision and prevention of elder abuse. Based on survey and focus groups, suggestions included ways to decrease social isolation among older adults by improving service outreach, provider training, and inter-organization communication. Discussion will outline gaps in service outreach and address future inter-organizational collaboration and strategies to prevent social isolation and elder abuse in rural communities.

